# A De Novo Arisen Case of Primary Adrenal Insufficiency in an Adolescent Patient With Crohn Disease: A Case report

**DOI:** 10.1097/MD.0000000000000818

**Published:** 2015-06-12

**Authors:** Yun Qiu, Ren Mao, Min-hu Chen

**Affiliations:** From the Department of Gastroenterology, First Affiliated Hospital of Sun Yat-Sen University, Guangzhou, People's Republic of China.

## Abstract

Several recent population-based studies have demonstrated that patients with inflammatory bowel disease are likely to have other autoimmune diseases. Here we describe the first de novo arisen case of primary adrenal insufficiency in an adolescent female patient with Crohn disease (CD).

A 17-year-old female diagnosed with stricturing colonic CD received the maintenance regimen of Remicade (infliximab) 5 mg/kg every 8 weeks following the standard induction regimen. She had an ileocecostomy due to acute small bowel obstruction at 1.5-year since the last infusion of Remicade. She was presented with skin hyperpigmentation of her face, neck, upper limbs, buccal mucosa and lips, which worsened when commenced on 6-mercaptopurine treatment for prophylaxis of postoperative recurrence. An increased adrenocorticotropic hormone (20.3 pmol/L, range 2–11) measurement was obtained. Radiography of the sella turcica region showed no signs of pituitary disease, or abnormality of bilateral adrenal cortex. Since serum aldosterone was below the reference range, more importantly, assessments for both antiadrenal antibodies and anti-21-hydroxylase antibodies were positive, she was then diagnosed as primary adrenal insufficiency. The symptoms improved after supplement of hydrocortisone.

This case highlights a rare immune-mediated comorbidity in an adolescent patient with CD. Recognition of a new pattern of autoimmune endocrine comorbidity enables clinicians to be alert about the possibility of concurrence of primary adrenal insufficiency with CD.

## INTRODUCTION

Several recent population-based studies have demonstrated that patients with inflammatory bowel disease (IBD) are much more likely to have autoimmune diseases such as arthritic autoimmune diseases or multiple sclerosis (MS) than patients without IBD.^1–6^ These data have led to speculation that IBD may share pathogenic pathways with some immune-mediated inflammatory diseases, possibly as a member of the larger autoimmune metadisease category.^7,8^ The concept of immune-mediated inflammatory disorders (IMIDs) has already been proposed.^7^ IMIDs are a group of diseases that involve inappropriate or excessive immune response, accompanied by dysregulation of the body's normal cytokine milieu.

Herein, we describe the first de novo arisen case of primary adrenal insufficiency in an adolescent female patient with Crohn disease (CD).

## CASE REPORT

A 17-year-old female patient was diagnosed with stricturing colonic CD in June 2010. Multiple biopsies were obtained for histopathological examination and mycobacterial culture. Histopathological examination revealed chronic nonnecrotizing granulomatous inflammation consistent with CD. Focal ulceration of the surface was noted. No epithelioid cells or the formation of tubercles were present and acid-fast staining was negative. Three experienced pathologists unanimously diagnosed CD. Tuberculin skin test and interferon-γ release assay further ruled out the possibility of intestinal tuberculosis.

**FIGURE 1:**
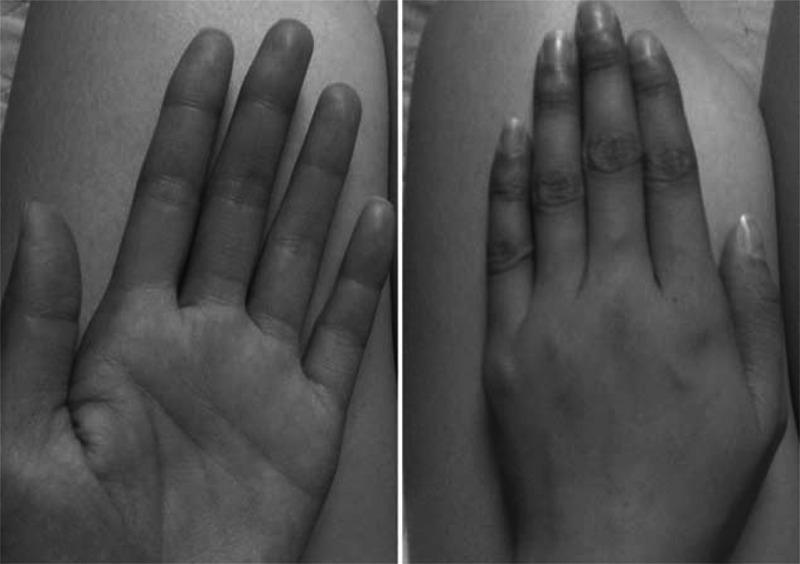
The skin hyperpigmentation of upper limbs.

After receiving the maintenance regimen of Remicade (infliximab) 5 mg/kg every 8 weeks following the standard induction regimen with an incomplete response, azathioprine was initiated at 50 mg/day. The drug was discontinued because of neutropenia. She was then commenced on methotrexate (MTX) 10 mg/week and the dose was incrementally increased to 15 mg/week over 5 weeks. During this time, her inflammatory markers remained high and her bowel symptoms showed no improvement (the Pediatric Crohn Disease Activity Index [PCDAI] ≥40). Severe active disease involving the transverse colon, descending colon, rectum, and sigmoid colon was found with colonoscopy and confirmed histologically. Furthermore, the CT enterography found segmental thickening of the cecum, ascending colon, terminal ileal, and the jejunum. After frequent episodes of subacute bowel obstruction responding to MTX, she had an ileocecostomy due to acute small bowel obstruction in the context of active medical therapy in July 2012. Sevoflurane-based epidural analgesias anesthesia was used and thoracic epidural analgesia was used for postoperative pain relief for 48 hours. Postoperatively, she presented with skin hyperpigmentation of her face, neck, upper limbs, buccal mucosa, and lips (Figure 1), which worsened after she commenced on 6-mercaptopurine (6-MP) 12.5 mg/day. Meanwhile, the patient complained of menstrual disturbance, poor appetite, intermittent abdominal pain, and diarrhea (PCDAI ≥30).

Her blood pressure was 115/60. She had a normal white cell count and an ESR of 52 mm in 1 hour (Westergren), biochemistry test revealed mildly increased levels of serum creatinine (120 μM, range 59–104 μM) and urea (455umol/L, range 140–360 umol/L). Laboratory tests revealed anemia (hemoglobin 95 g/L, range 120–160 g/L). Iron studies revealed a low serum iron level (59.2 μg/dL, range 65–175 μg/dL), low total iron-binding capacity (273 μg/dL, range 280–430 μg/dL), and an elevated ferritin level (669 ng/mL, range 24–336 ng/mL). Serum electrolytes (plasma sodium, potassium, chlorides), liver function, blood glucose, amylase, and lipase were all within normal limits.

**FIGURE 2:**
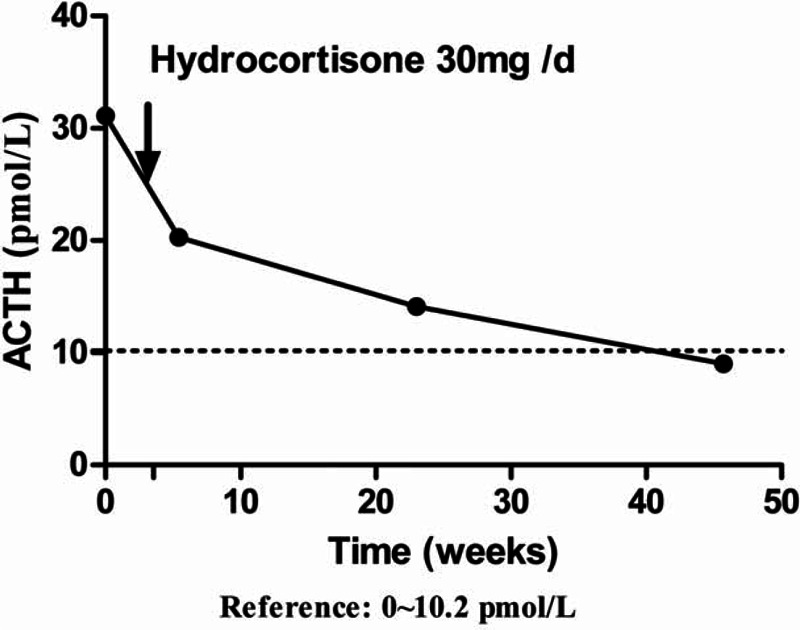
The change of ACTH since the replacement therapy (reference: 0∼10.2 pmol/L). ACTH = adrenocorticotrophic hormone.

The 6-methyl-mercaptopurine level at the time was in the nontoxic range and the 6-thioguanine nucleotide level was subtherapeutic. The thyroid endocrine function test was normal (Table 1). Adrenocorticotrophic hormone (ACTH, 20.3 pmol/L, range 2–11 pmol/L), serum cortisol (morning 70 pg/L, range 100–200 pg/L; evening, 31 pg/L, range 50–100 pg/L), urinary cortisol (4.1 μg/dL, range 4.3∼176 μg/dL), and serum aldosterone (36 pmol/L, reference range 140–560 pmol/L) measurements were determined by liquid chromatography-tandem mass spectrometry (LC-MS/MS). The circulating ACTH concentration was too low to be characteristic of adrenal disease, and too high to be typical of pituitary disease. Contrast-enhanced axial CT scans of pituitary and bilateral adrenal glands were done with the patient in a prone position. Radiography of the sella turcica region showed no signs of pituitary disease, or abnormality of bilateral adrenal cortex. The impaired ability of the adrenal cortex to respond to ACTH is readily demonstrated by the standard short corticotrophin test,^9^ which involves measurement of serum cortisol before and after 30 or 60-minutes intramuscular injection of 250 μg ACTH. This challenge did not evoke any further increase in serum cortisol. Assessment for antiadrenal antibodies and anti-21-hydroxylase antibodies was positive, and antinuclear antibodies (ANA) and antidouble stranded DNA (anti-dsDNA) autoantibodies were negative both prior to and after the infusion of infliximab. She was then diagnosed as primary adrenal insufficiency (or Addison disease). Treatment with hydrocortisone 30 mg per day was initiated with a good response (Figure 2). These symptoms improved after the supplement of hydrocortisone along with the cessation of 6-MP. She restarted on MTX 15 mg weekly with concomitant Salofalk tablets (3 g per day) after the confirmation of postoperative recurrence (Rutgeerts scores, i3). During the following 6 months’ treatment, she has been maintained in reasonable health, mild anemia persists despite a course of intramuscular iron.

**TABLE 1:**
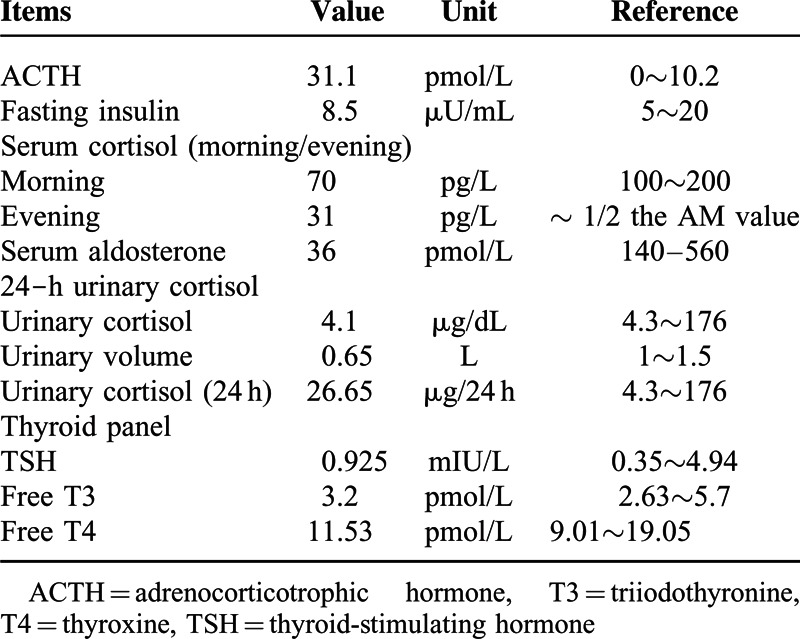
Results of Endocrine Function Test

The study was approved by the Clinical Research Ethics Committee of the First Affiliated Hospital of Sun Yat-sen University. Informed consent was obtained.

## DISCUSSION

Biological agents are being increasingly used in the treatment of CD, with a growing number of reports about the paradoxical induction of autoimmune processes.^10–12^ Anti-TNF therapy may lead to paradoxical inflammatory skin (eczema and psoriasis) and joint (polyarthralgia) or ocular (uveitis and scleritis) manifestations,^13^ demyelinating central nervous system disorders, sarcoidosis, development of ANA, and in rare cases, lupus.^14^ The occurrence of autoimmunity during anti-TNF treatment may not be related only to the increased apoptosis but also to unbalance of inflammatory pathways (paradoxical inflammation). For example, it has been recently reported that psoriasiform skin lesions characterized by Th17 and Th1 cell infiltrations developed in nearly 5% of anti-TNF-treated patients with IBD, and these patients could be effectively treated with anti-IL-12/IL-23 antibody.^15^

The humoral response induced by infliximab was restricted to ANA and anti-dsDNA autoantibodies, which generally did not lead to clinical signs of autoimmunity.^11^ The latest update of a Spanish registry^16^ reported more than 800 cases of autoimmune diseases were secondary to biological therapies, including a wide variety of both systemic and organ-specific autoimmune processes. The majority of cases appeared between 1 month and 1 year after initiation of the biological agents. In this case, although a causal relationship is not conclusive, the apparent temporal association should raise enough concerns.

One possible explanation for the development of autoimmune disease secondary to anti-TNFs therapy is that the release of autoantigens triggered by increased apoptosis will further induce the formation of autoantibodies against cytoplasmic and nuclear compounds (ie, ANA and dsDNA), but not specific organs due to the lack of organ-specific TNF-α receptors.^11^ This may partially explain the absence of autoimmune endocrine diseases. Furthermore, the larger pool of autoantibodies of the IgM isotype observed during infliximab treatment instead of IgG, which have a well-known pathogenic effect, could be due to a higher production of natural autoreactive IgM.^17^ Due to negative for ANA and anti-dsDNA autoantibodies prior to and after infusion of infliximab, the possibility of anti-TNF-α treatment induced adrenal insufficiency was ruled out.

Patients with IBD are much more likely to have autoimmune diseases.^1–6^ A recent study included 737 children with CD (1997 controls) and 488 with UC (1310 controls) demonstrated that CD was associated with a higher prevalence of RA (odds ratio [OR] 15.7), lupus (OR 41.0), and hypothyroidism (OR 2.9).^18^ This report added to the current evidence of an elevated risk of autoimmune disease in IBD.^1–5^ An underlying pathogenic mechanism based on a dysfunctional immune system is clearly suggested by these data.^18^ The identified risk loci shared by SLE and other autoimmune disorders (ie, Behçet disease and IBD) with genome-wide association study (GWAS) already suggested the existence of common immunologic mechanisms. IBD may share pathogenic pathways with other immune-mediated inflammatory diseases, possibly as a member of the larger autoimmune metadisease category.^7,8^

To the best of our knowledge, no relationship between primary adrenal insufficiency and CD has been described before. Adrenal insufficiency is classified as primary and secondary. In our case, there were no significant low blood pressure episodes during surgery. Thoracic epidural analgesia was used temporarily for postoperative pain relief. The reports of effects of opioids on ACTH are still conflicting. In a study on the chronic effects of intrathecal opioid administration (with a mean duration of 26.6 ± 16.3 months), only 1 patient developed symptomatology of an addisonian crisis during an episode of important fever due to pneumonia.^19^ Scarce case reports have documented adrenal insufficiency after chronic oral^20^ or transdermal^21^ opioid administration but not acute intrathecal administration. In our case, the patient received intrathecal administration of opioids for no more than 48 hours, so the presented adrenal insufficiency should probably not be due to side effects of narcotics. Abrupt withdrawal of corticosteroid therapy could cause adrenal insufficiency secondary to suppression of the hypothalamic-pituitary-adrenal (HPA) axis. As demonstrated in Christy et al study,^22^ the use of corticosteroids for ≥2 weeks could lead to an impaired responsiveness of adrenal glands for ≥16 months. With regard to our patient, she received an induction regimen with anti-TNFs therapy when diagnosed, and then maintained on MTX due to intolerance to thiopurine before operation without receiving glucocorticoids during the whole disease course.

In summary, we ruled out the possibility of secondary adrenal insufficiency in this patient based on the following reasons. First, no positive findings were detected by CT scans of pituitary and bilateral adrenal glands. Secondly, the patient did not receive exogenous glucocorticoid treatment during the whole disease course including perioperation. Thirdly, there were no signs of iatrogenic injury. And most importantly, the antiadrenal antibodies and anti-21-hydroxylase antibodies were positive, which are diagnostic for autoimmune aetiology.

It is reported that about 50% of patients with autoimmune Addison disease have polyendocrine syndromes.^23,24^ Early in 1958, Prunty et al presented a case of CD with features suggesting Addison disease.^25^ Unluhizarci et al described a patient with Addison disease who was also diagnosed as collagenous colitis and successfully treated with sulfasalazine.^26^ In our case, we have ruled out the possibility of autoimmune polyglandular syndrome based on the normal thyroid function (thyrotropin level is normal and autoantibodies against thyroid peroxidase were absent) and normal fasting plasma glucose.^23^

Herein, we presented a new immune-mediated comorbidity in an adolescent female patient with CD. The finding suggests that there may be common etiological mechanisms between these immune-mediated inflammatory diseases, which may help further define IBD-related comorbidities.
